# Innovative healthcare solutions: robust hand gesture recognition of daily life routines using 1D CNN

**DOI:** 10.3389/fbioe.2024.1401803

**Published:** 2024-07-31

**Authors:** Naif Al Mudawi, Hira Ansar, Abdulwahab Alazeb, Hanan Aljuaid, Yahay AlQahtani, Asaad Algarni, Ahmad Jalal, Hui Liu

**Affiliations:** ^1^ Department of Computer Science, College of Computer Science and Information System, Najran University, Najran, Saudi Arabia; ^2^ Department of Computer Science, Air University, Islamabad, Pakistan; ^3^ Department of Computer Sciences, College of Computer and Information Sciences, Princess Nourah Bint Abdulrahman University, Riyadh, Saudi Arabia; ^4^ Department of Computer Science, King Khalid University, Abha, Saudi Arabia; ^5^ Department of Computer Sciences, Faculty of Computing and Information Technology, Northern Border University, Rafha, Saudi Arabia; ^6^ Cognitive Systems Lab, University of Bremen, Bremen, Germany

**Keywords:** hand gesture recognition, multi-fused features, deep learning, convolutional neural network, healthcare

## Abstract

**Introduction:**

Hand gestures are an effective communication tool that may convey a wealth of information in a variety of sectors, including medical and education. E-learning has grown significantly in the last several years and is now an essential resource for many businesses. Still, there has not been much research conducted on the use of hand gestures in e-learning. Similar to this, gestures are frequently used by medical professionals to help with diagnosis and treatment.

**Method:**

We aim to improve the way instructors, students, and medical professionals receive information by introducing a dynamic method for hand gesture monitoring and recognition. Six modules make up our approach: video-to-frame conversion, preprocessing for quality enhancement, hand skeleton mapping with single shot multibox detector (SSMD) tracking, hand detection using background modeling and convolutional neural network (CNN) bounding box technique, feature extraction using point-based and full-hand coverage techniques, and optimization using a population-based incremental learning algorithm. Next, a 1D CNN classifier is used to identify hand motions.

**Results:**

After a lot of trial and error, we were able to obtain a hand tracking accuracy of 83.71% and 85.71% over the Indian Sign Language and WLASL datasets, respectively. Our findings show how well our method works to recognize hand motions.

**Discussion:**

Teachers, students, and medical professionals can all efficiently transmit and comprehend information by utilizing our suggested system. The obtained accuracy rates highlight how our method might improve communication and make information exchange easier in various domains.

## 1 Introduction

The study of hand gestures is becoming a growingly popular discipline among various aspects of human activity recognition (HAR) (Liu, H et al. (eds), 2023). The major purpose of the study of these gestures is to reform the gestures, both static and dynamic, that arise in our environment (Hu, S. et al., 2022) The studies of gestures are not only interesting but also very useful in aiding psychology, anthropology, sociology, cognitive science, and communication (Mo, H et al., 2020; Rezae, K. et al., 2021). Hand gestures are used to express feelings in multiple ways, give clues to the understanding of characters, and reduce anxiety and stress. Hand gestures are an excellent substitute for communication with deaf people; they tell us what is going on inside their heads because we are unable to communicate verbally (Maritta, A. et al., 2021). To teach and learn efficiently, teachers need to be able to express their ideas clearly and effectively. To be able to do that, they must first understand the common gestures used by students and teachers. This is true for any field of learning. In the context of online learning, teachers frequently encounter difficulties while attempting to successfully communicate with students using sign language. It may be difficult to communicate complicated or difficult concepts using current solutions since they are unable to sufficiently track and recognize hand movements (Sundaram and Chaliapin, 2020; Zhu, Y. et al., 2022; Wang, N. et al., 2022; Rehman and Ullah, 2022). By creating a dynamic hand gesture tracking and recognition system that enables smooth communication between instructors and students in an online learning environment, the proposed research seeks to overcome these shortcomings.

In the medical field, hand gestures are very important, especially when communicating with patients or medical specialists (Tripathi and Vishwakarma, 2019; Gochoo and Jalal, 2021; Wang, K. et al., 2023; Cai, L. et al., 2023). Nevertheless, the precision and accuracy of current techniques for hand gesture tracking and detection in medical settings may be inadequate, impeding efficient communication (Zhang, R. et al., 2023; Zhao, S. et al., 2024) and patient care. In order to address these issues, this study suggests a novel method that allows medical professionals to interact with patients by using hand gestures to convey discomfort, ask for assistance, or show hunger. Understanding how hands are used in different medical fields can help people in the future when they are dealing with more advanced physiology. Hand gestures can be defined as the physical interaction of objects in the hand space. It is important to understand these gestures in order to become a better and more efficient person (Anastasiev, A. et al., 2022; Grant and Flynn, 2017). In the medical field (Khan, D. et al., 2024), there are many different types of hand gestures. For example, hand gestures in the physical therapy world are used to control the person’s position when trying to treat them (Gochoo and Jalal, 2021).

In this research paper, we have proposed a dynamic approach to 3D hand gesture tracking and recognition for the e-learning platform (Yu, J. et al., 2021) to help teachers communicate with students through sign language during class and also keep track of their class notes, help students remember the answers to their questions, and also help them understand complex or challenging concepts. On the contrary, this system also helps medical specialists communicate with their patients through various hand gestures like pain, help, and hunger (Hou, X. et al., 2023; Shen, X. et al., 2022; Jiang, H.et al., 2023). For the proposed system, two benchmark datasets are selected, Indian Sign Language (ISL) and WLASL, for system training and testing. The system is dependent on six major steps. i.e., 1) pre-processing: the hand gesture dataset videos are converted into frames, and then a fixed dimension is set to resize the frames, and noise is removed from the frames. 2) Hand detection is conducted using background modeling using the Gaussian mixture model (GMM) (Liu, H. et al., 2021; Hartmann, Y. et al., 2022) and CNN (Cao et al., 2024) for bounding box formation. 3) Skeleton mapping is conducted for point-based feature extraction, where the hand skeleton is mapped on the entire hand using SSD tracking based on the landmarks plotted on the hand. 4) Feature extraction: we have followed two approaches for feature extraction: point-based feature extraction and full-hand coverage feature extraction. For both of these approaches, we have used some techniques, which be read about in Section. 5) Optimization: this is conducted to obtain more precise and accurate results. We have used the population-based incremental learning (PIL) technique. 6) Classification: at last, the optimized set of features is passed to the 1D CNN classifier for classifying the dataset classes.

The major contributions and highlights presented in this paper are summarized as follows.

We proposed a robust hand detection technique that promises to give the best results of hand detection using background modeling using GMM and bounding box formation using the CNN technique. We have used both point-based and full-hand coverage-based features to better train our model. Population-based incremental learning optimization is used for the first time in hand gesture tracking and recognition and gives us promising optimization results. For classification, we have adapted 1D CNN, which gives promising classification results on videos.

The rest of the article is arranged as follows:

Section 2 presents the literature review. Section 3 describes the methodology of our proposed system. Section 4 provides a performance evaluation of our proposed approach on two benchmark datasets and also a comparison and discussion. Finally, in Section 5 we conclude the paper and outline the future directions:

• Development of robust denoising techniques tailored for signal and audio sensor data, enhancing activity recognition accuracy.
• Extracting novel features for detecting human localization information.
• Development of a hybrid system that combines machine learning and deep learning features to further improve activity recognition performance.
• Furthermore, a comprehensive analysis was performed on well-known benchmark datasets, which feature diverse human actions and advanced sensors.

## 2 Literature review

Nowadays, for hand gesture tracking and recognition, different computer vision approaches have been proposed by researchers. In this section, we categorize the related work into two subsections, the first section describes the recognition of hand gestures for student learning; however, the second subsection describes hand gesture recognition used by medical specialists to communicate with the staff and the patients.

### 2.1 Hand gesture tracking and recognition for student learning

Many researchers have worked on different models to track and recognize hand gestures for student learning. They have presented ways to recognize sign words for communication between the tutor and student. In addition, applications are designed via computer vision to help in a particular domain; however, hardware systems are also presented to solve the issue.

Boruah, B.J. et al., 2021) used three approaches for hand tracking and recognition. First, the hand palm detection is conducted by using a trained palm detector model. Second, regression is used to localize the 21 landmarks on the entire hand. Third, a projected hand skeleton is used to train a model to classify the hand gestures. At the end, MediaPipe is used to classify hand gestures for controlling various objects. They have used a vision-based system for their model. The use of expensive equipment for system design was neglected. The built-in models were used for better recognition accuracy. The system has only used six classes for controlling the 3D objects, which are not sufficient. The system should be trained on more classes to better handle the objects. Erazo, O. et al.( 2017) designed a hand gesture recognition system to increase the interactivity of the students during class lectures. The system was designed for the students to interact with the screen to perform experiments. These gestures include hold, tap, pull, swipe, release, draw, wave, and grip. The gestures are dependent on the screen that recognizes the gestures. The viability of implementing touchless hand gestures in lectures is to encourage and facilitate student involvement to increase participation. THG-based applications were proposed for gesture recognition. Users cannot interact with the screen beyond a certain distance threshold. Second, the model trained on seven classes was not enough to fully operate the screen. Students who are handicapped cannot use this system to perform experiments. A hand gesture recognition system (Xiao, Z. et al., 2023; Zhao, X. et al., 2024) that is used for learning the 3D geometry in school has been developed. The paper is based on two technologies; AR and hand gesture recognition. The students can understand the basic concepts of 3D geometry and also to construct different 3D geometrical shapes in 3D space using VR, whereas the hand gesture recognition can help the students operate the 3D geometrical shapes and construct them in 3D space using different hand gestures. They suggested software that would address certain challenges in geometry teaching and give students an easier approach to study geometry by fusing augmented reality (AR) and recognition of hand gesture technology. The model was trained in very few classes, which is not enough to learn geometry. However, the response rate of the intuitiveness (very easy) was also low. The system was not good for handicapped students. The purpose of this research (Liu, N. et al., 2003) is to recognize the alphabet using hand gestures. For that, the author used the YUV skin segmentation technique to detect the hand movements. Different morphological operations were applied to remove the noise from the images. Then, the CamShift algorithm is used to track the hand movements. Features are extracted for further classification of the alphabets. Hand centroid is calculated, and the HMM algorithm is used to recognize the 26 alphabets. The proposed system provides a hardware-free model to recognize all alphabets. They trained their own dataset for proposed architecture training and testing. The YUV hand detection technique does not always yield promising results when the skin color of the person varies. Second, many alphabet trajectories have a large similarity index. The system is unable to identify the correct alphabet, for example, letter C is confused with G and O. Bhowmick, S. et al. (2015) proposed a system to recognize the alphabets using hand gestures. The author used a webcam to record the videos at a resolution of 720 × 480 at 40 fps. The hand is segmented using the HSV + YCbCr skin segmentation technique. The hand is segmented to exclude the background and find the region of interest. Features are extracted to find their orientation, gesture trajectory length, and velocity and acceleration. Then, the classification is performed using the MLP-ANN and FTDNN. They propose a system, especially considering the needs of deaf and mute people. A deep neural network is used in the system to attain higher accuracy. They trained all alphabets by extracting gestures from the background using a simpler technique to reduce computation costs. The system does not give satisfactory recognition results for the alphabets that look similar. For example, alphabets like C, G, and O or E and F. (Zhu, M. et al., 2023) in their research presented a novel gesture recognition method named DDF-CT. It creates range-time and range-angle maps by using radar signals to extract range, angle, and Doppler information. To improve temporal connection learning and feature extraction, the approach integrates deformable convolution and inter-frame attention methods. The accuracy of 98.61% is demonstrated by the experimental findings, with 97.22% accuracy even in new surroundings. In terms of accuracy and robustness, the DDF-CT method performs better than current techniques for hand gesture recognition.

### 2.2 Hand gesture tracking and recognition systems for medical specialists

Various research studies have devoted their time and energies for developing hand gesture recognition systems that can help medical specialists communicate with the patients and staff (Xu et al., 2016; Islam, M.N. et al., 2022; Wan and Chan, 2019). In Gedkhaw and Ketcham (2022), an IoT system to recognize the message of the patients using hand gestures is designed. The Haar cascade algorithm is used to detect the hand, and principal component analysis (PCA) is used with the histogram oriented gradient (HOG) to achieve better accuracy. The system recognizes eight classes which are need to relax, pain, hunger, thirst, meet a doctor, take medicine, go to toilet, and please rub the body dry. The designed model is made up using simple techniques. They made the IoT system which recognizes messages of patients effectively. The proposed method was not effective in different cases, and the model needs improvements for better recognition. The author (Lamb and Madhe, 2016) proposed a system to control the movement of bed for accidental patients, old age patients, and paralyzed persons. The patient uses certain hand gestures to move the bed up, down, left, and right according to their comfort level. At first, some pre-processing is conducted to remove the background and extract the hand (Liu, H. et al., 2022; Fu, C. et al., 2023). Then, the wavelet decomposition is used to extract the shape features, and at last Euclidean distance is used as a classifier. They used the existing bed and updated the movements by using microcontrollers and sensors. The bed only moved up and down; however, they added two more positions and also tracked down the patient’s fall. The proposed method was not effective in different cases, and the model needs improvements for better recognition. The purpose of this research (Haider, I. et al., 2020; Liu, H. et al., 2022) is to facilitate communication in mute persons and make it easy for them. In this system, a KINECT image base sensor is used to sense the hand gesture of the person and then decode that hand gesture into meaningful audio output to communicate with the person. They build a device to interpret gestures. The device is user-friendly and cost-effective. The device translates the hand gesture and provides audio sound as interpretation (Cao and Pan, 2024; Xue, Q. et al., 2023). A large dataset is used to train the decoder in recognizing the hand gesture and interpreting correctly as to what the person is saying. The system is not reliable enough to correctly decode every hand gesture. Fayyaz, S. et al. (2018) designed a system to control the movement of bed using hand gestures. This system is based on image processing techniques. First, the hand is detected by applying the HSV color space on video frames. Then, the hand contours are extracted using the erosion and dilation filter and Pavlidis algorithm. The palm central point is calculated through the Skyum algorithm. Then, the position of the fingers is calculated through the Gram algorithm. The machine algorithm is used to recognize the hand gestures, and a DC motor is attached with the Arduino UNO kit and bed to control the movement. A webcam is used to create the computer vision system. Simple hardware components are used to build the system, which reduced the high equipment cost. The execution time to recognize the hand movement and move the bed is significantly high with respect to other models. The proposed model is developed to communicate with the deaf community using hand gestures. At first, the bicubic technique is used to resize the original images. Then, the low-pass filtration is used to remove the noise. The feature vector implementation SIFT algorithm is used, and for vector comparison, Euclidean distance is used, a proper model for deaf people using computer vision, rather than using color markers or gloves. Light intensity improves the result accuracy, but more light intensity causes blurring of the image and affects the output result. The quality of the image in the database and the input image should also be moderate so that the feature vector can be matched easily, and a decrease in image quality can result in no match. A convolutional neural network (CNN) is used by Alonazi, M, et al. (2023) to recognize hand motions. Following the detection of the hand gestures, features are retrieved via a competitive learning technique known as neural gas. Furthermore, locomotion thermal mapping is carried out in order to improve the feature extraction procedure even more. Fuzzy feature optimization is used to compute a feature vector following feature extraction. Fuzzy logic techniques are used in this procedure to optimize the feature vector representation.

## 3 Materials and methods

### 3.1 System methodology

**FIGURE 1:**
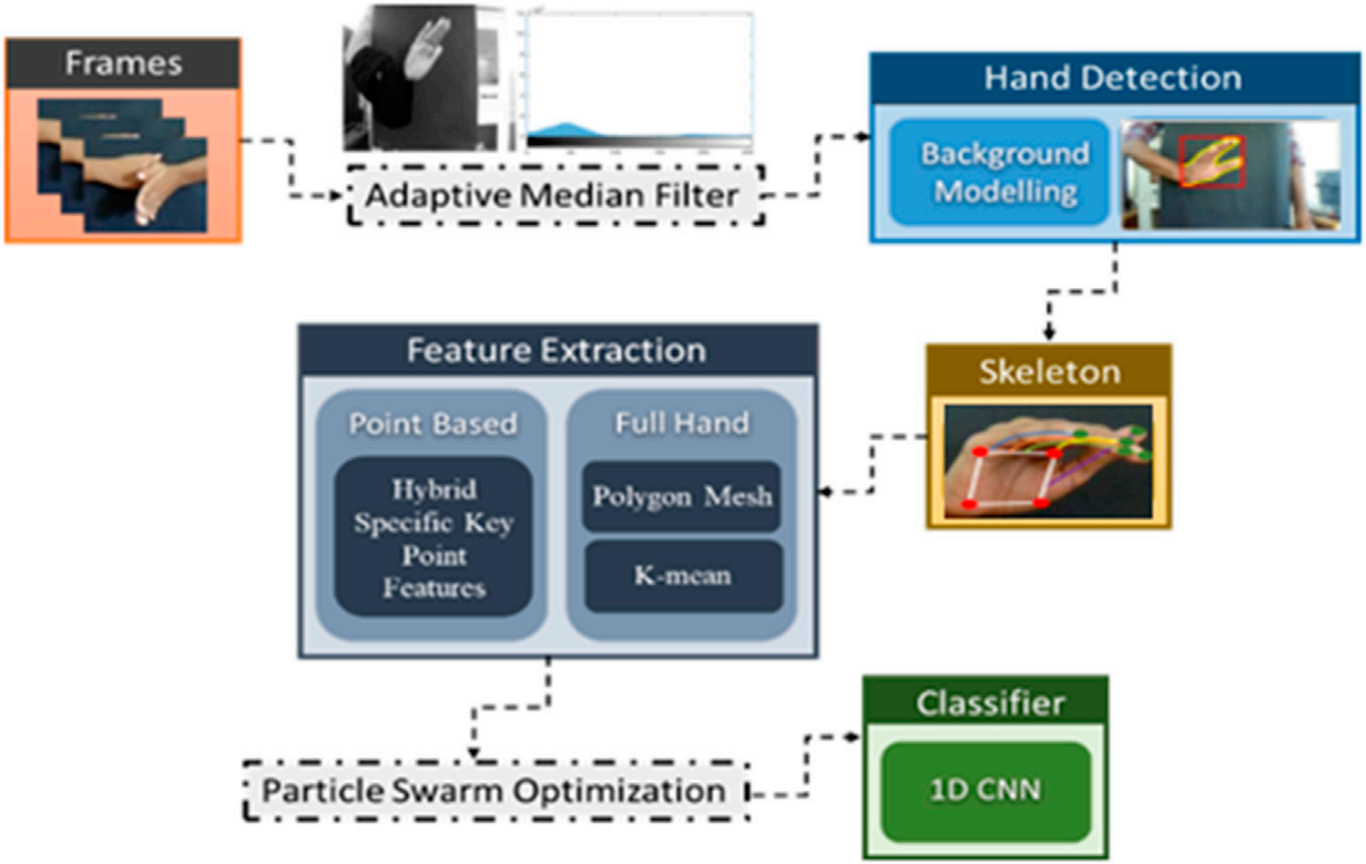
Proposed system architecture using different hand gestures.

In this paper, we have proposed a dynamic approach to hand gesture tracking and recognition to help teachers, students, and medical specialists convey their information in a better way. Our approach is subdivided into six modules. Initially, the videos are converted into frames, and pre-processing is conducted to enhance the quality of the frames using the adaptive median filter (AMF). Furthermore, hand movements are detected by background modeling and the CNN method. After that, hand skeleton mapping is conducted using SSD tracking. The next step is to extract the features for better training of the model. For that, we have used point-based and full-hand coverage techniques. However, the population-based incremental learning optimization algorithm is used to get the most accurate results possible. At last, a recurrent neural network (RNN) classifier is used to recognize the hand gestures. Figure 1 depicts the overall structure of our proposed hand gesture tracking and recognition model. In the following subsections, the details of each of the aforementioned modules are explained.

### 3.2 Preprocessing

**FIGURE 2:**
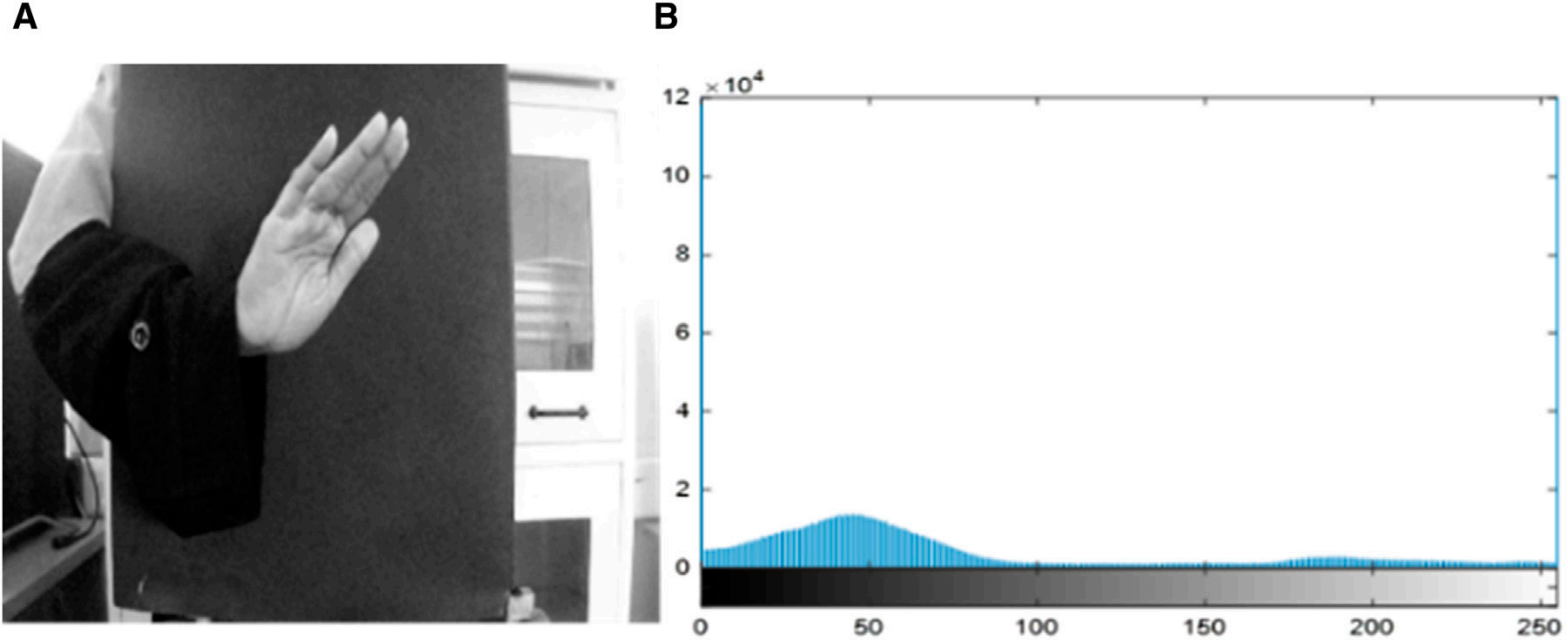
Pre-processing on call gesture. **(A)** Filtered and enhanced image via AMF and **(B)** histogram of filtered and enhanced images.

In the first phase, the static RGB video is converted into frames. Through the AMF, frames are passed to effectively exclude noise and distortion, which result in smooth edges. The AMF filtration is conducted in two stages. At first, each pixel of the original frame is compared with the neighboring pixel using a certain threshold to detect noise (Zhao, P. et al., 2024; Miao, R. et al., 2023). Then, it classifies the pixels below a certain threshold as noise based on spatial processing. The noised pixels of the frame are known as impulse noise, which is not similar compared with the other neighborhood pixels. After the noise labeling test, the pixels passed through it are replaced by the median pixels. On the filtered images, AMF histogram equalization was performed to adjust the contrast of the image using Eq. 1 (Zhao, Y. et al., 2024).

$$s_{k}=T\left(r_{k}\right)=\left(M-1\right)\sum_{j=0}^{k}p_{r}\left(r_{j}\right)\;H\left(f\right),$$ (1)

where s denotes the output intensity level, k = 0, 1, 2, …, $\left(M-1\right)$ , and r denotes input image intensities which need to be processed. r = 0 represents black, and r = M−1 represents white, as r is in the range [0 – ( M−1 )]. $p_{r}\left(r\right)$ represents the probability density function (PDF) of r , where in $p_{r}$ , subscript of p was used to indicate the PDF of r . By mapping each pixel on the input image with intensity $r_{k}$ into a corresponding pixel with level $s_{k}$ in the output image, a processed output was achieved using Eq. 1, as shown in Figure 2.

### 3.3 Background modeling

**FIGURE 3:**
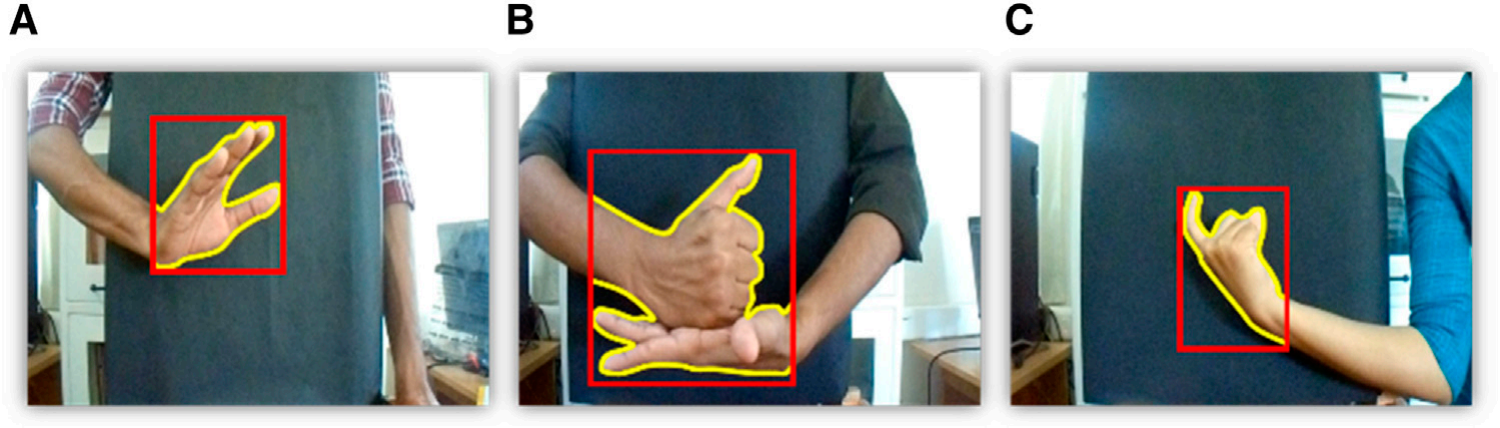
Polygon and bounding box obtained using the proposed method over ISL dataset gestures **(A)** call, **(B)** help, and **(C)** hot.

By using the proposed model, an accurate shape descriptor estimation for the hand gesture is achieved. At the initial stage of our detection framework, we are looking for region proposals based on the variation in image intensities. To get better accuracy, we approach each region proposal from per-pixel analysis first, which then forms a bounding polygon and eventually the bounding box as shown in Figure 3. We have adapted GMM (Khan and Kyung, 2019) to robustly distinguish the foreground pixels from the constantly updated background pixels. Suppose that the RGB components of the pixels are independent and identically distributed random variables in the RGB color space, so we take Gaussian models G per channel of the pixel over time. Let $M_{p,c}^{k}=\left(\mu_{p,c}^{k},\sigma_{p,c}^{k}\right)$ be the *k*th distribution of the channel c≤C at pixel p≤Ω , the model is assigned by a pixel, and providing a new frame $X_{i}$ if and only if using Eq. (2) (Oudah et al., 2020).

$$\begin{array}{ccc} | & X_{i}^{c}\left(p\right)-\mu_{p,c}^{k} & | \end{array}<m\;.\;\sigma_{p,c}^{k},$$ (2)

where $X_{i}^{c}$ is the *c*th slice of the image $X_{i}$ and m is the threshold. Practically, we have selected $m\in \left\{1.5,3.1\right\}$ for the best results generated by our model. The posterior distribution for the *k*th model is updated by the assignment (Li et al., 2022). If the closest Gaussian distance $X_{i}^{c}\left(p\right)$ is achieved by a model normalized by its standard deviation is the background model in $X_{i}^{c}$ whereas p is considered to be the foreground image pixels.

### 3.4 Hand movement detection

The CNN is applied to $X_{i}$ to get the set of observations denoted by $\hat{Z_{i}}$ . For each $\hat{z\;}$ $\epsilon \;\hat{Z_{i}}$ −1. The optimal result is found based on the previous observation $\hat{Z_{i}}$ −1 using Eq. (3) (Merad and Drap, 2016; Chahyati and Arymuthy, 2020; Pradeepa and Vaidehi, 2019; Gadekallu et al., 2022; Zhang et al., 2020; Li et al., 2022).

$$\underset{\;\hat{z}\;\epsilon \;{\hat{Z}}_{i}\;-1}{\mathrm{argmax}}\left\{v_{c,i-1}.\;\left(\Gamma_{i-1}\left(z_{c}\right)-\Gamma_{i}\left(\hat{z}\right)\right),\right.$$ (3)

where $v_{c,i}$ is the binary term used to indicate whether object $w_{c}$ is observed in the *i*th frame. $\tau \left(z\right)$ is the normalized zero mean of the 1−σ image patch covered by $\beta \left(z\right)$ . The bounding box is formed by z and * shows the correlation operator. The association is then verified using a distance check. If the value of $z_{i}$ is too far from $\hat{z}$ in, then the correspondence $Z_{c}↔\;\hat{z}$ is rejected.

**FIGURE 4:**
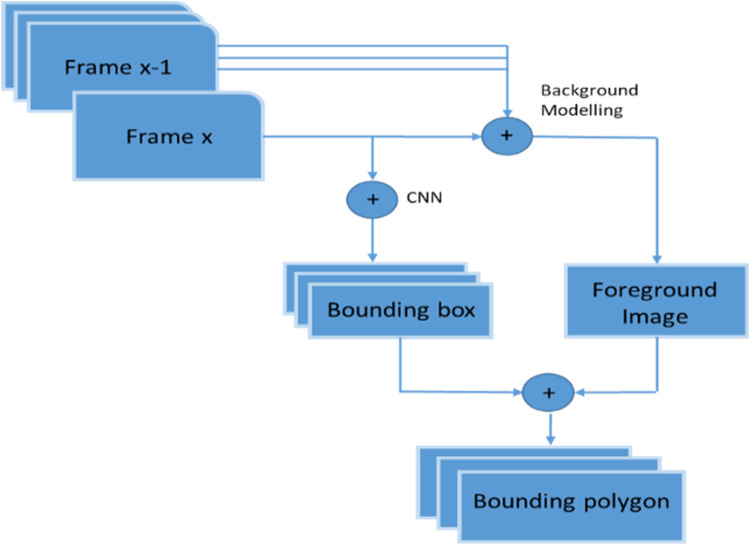
Overall hand detection model.

For the pixels $\hat{z}$ which do not match the previously tracked pixels, a new entry is created and appended to $\hat{Z_{i}}\;-1$ by making a new observation set (see Figure 4).

### 3.5 Hand skeleton mapping

**FIGURE 5:**
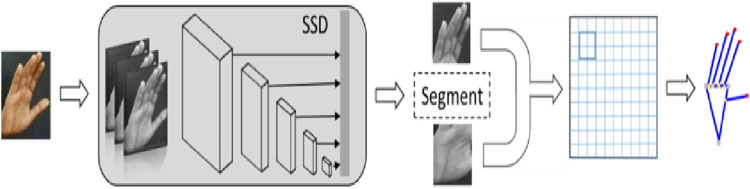
Model of hand skeleton mapping.

**FIGURE 6:**
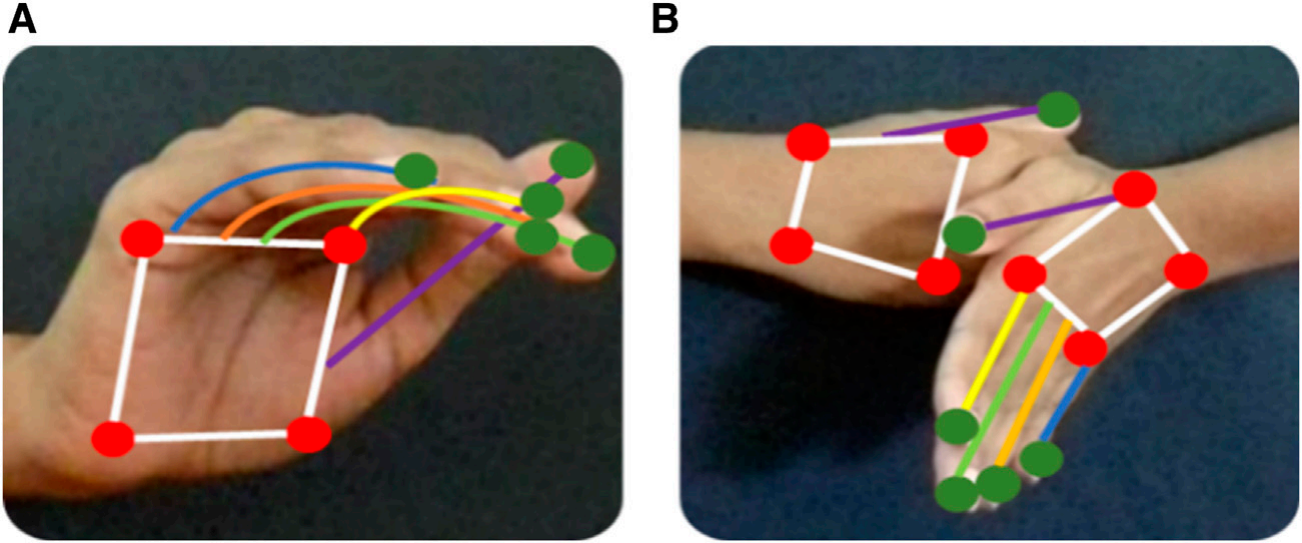
Skeleton mapping on ISL dataset gestures **(A)** pain and **(B)** accident.

The first and foremost step in hand skeleton mapping is the localization of the hand. For this, we have used the single shot multibox detector (SSMD) to detect the palm, excluding the fingers. The palm is bounded by a blob. The palm region is converted to binary, and the four-phase sliding window is moved to detect the extreme left, right, top, and bottom points. The next step is the localization of the fingers. Again, we have used a pre-trained SSMD to detect the fingers, excluding the palms of the hands. The four-phase sliding window is moved to identify the extreme top, bottom, left, and right points. As a result, we have obtained five points on the fingers and four points on the palm (Khan, M.U.K. et al., 2018; Zhou, L. et al., 2021; Yimin, D.O.U. et al., 2019; Nawaratneand et al., 2019; Chen et al., 2018). Figure 5 shows the overall hand skeleton mapping model, and Figure 6 shows the mapping result on the ISL dataset.

### 3.6 Multi-fused features

Feature engineering is essential for human gesture and activity recognition (Hartmann, Y. et al., 2023). In this section, we used key point-based feature extraction methods using specific and full-hand landmarks. It is observed from our testing that during the hand movement, forming of different hand gestures in the video gives more precise results as compared to the texture-based feature. The reason is that when hand movement occurs, the key landmarks located on the palm and the fingers show significant change. We have used 1) hybrid-specific key point features, 2) polygon meshes, 3) K-mean ellipses, and 4) co-occurrence generation techniques for feature extraction. These topics are further discussed in the next sections. Algorithm 1 describes the overall feature extraction techniques.

Algorithm 1 3D hand gesture feature extraction.

Input: F $=\left\{H^{1},H^{2},.\;.\;.\;.,H^{z}\right\}$

//where F is the set of video frames.
Output: Normalized feature vectors $V^{1},V^{2},.\;.\;.\;.,V^{z}$

Feature vectors← get_window_size()
Overlap_time ← get_overlap_time()
For HandComponent in [x,y,z] do
Hand_Feature←get_window(hand features)
//Extracting point base features
Hybrid_keypoint_features←Extract_hrbrid_features(Hand_Feature)
Polygon_meshes←Extract_polygon_meshes(Hand_Feature)
Kmean_ellipsoids←Extract_kmeanEllipsoids_features(Hand_Feature)
cooccurance←Extract_cooccurance(Hand_Feature)
festure_vectors←GetfeatureVectors(Hybrid_keypoint_features, Polygon_meshes,Kmean_ellipsoids, cooccurance) feature_vectors.append(feature_vectors)
end for
feature_vectors←Normalize(feature_vectors)
return feature_vectors

#### 3.6.1 Hybrid-specific key point features

**FIGURE 7:**
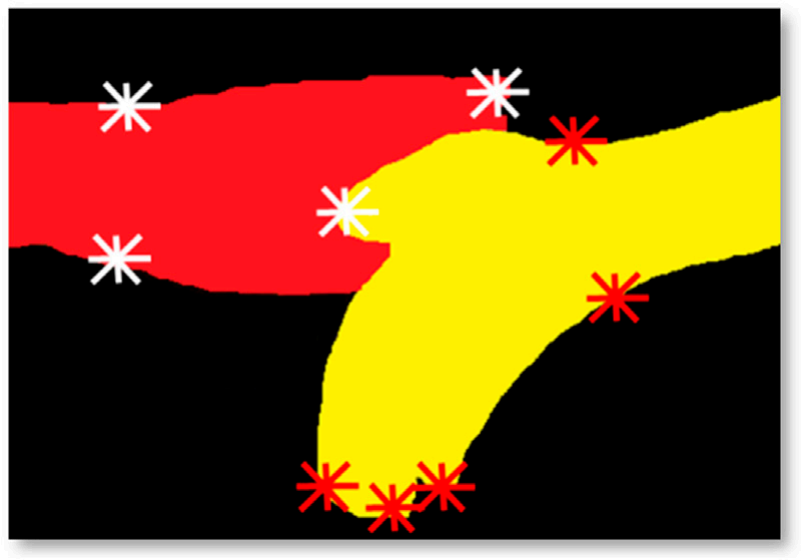
Hybrid-specific key marked points on accident gesture.

**FIGURE 8:**
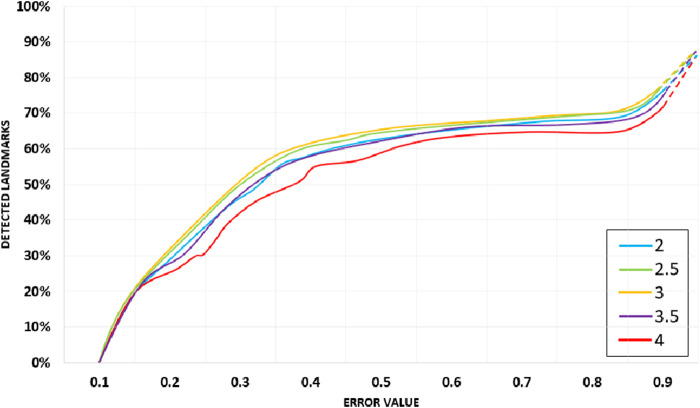
Hybrid-specific key graphical representation.

In this section, we have explained the hybrid key point-based features using the key landmark points of the hand. At first, the hand silhouettes are represented with different colors, and their boundary points are stored. Then, the center point of the hand silhouette is calculated by accumulating the area inside the silhouette (Jana, A. et al., 2022; Minaee et al., 2021). To locate the interacting fingering with the palm or other fingers during different hand gestures, the topmost, left, right, and bottom boundary pixels are marked with a point, as shown in Figure 7. The distance between the interacting hand fingers or the palm is calculated as Eq. 4.

$$h\left(f_{1},f_{2}\right)=\sqrt{{\left(f_{1}x-f_{2}x\right)}^{2}+{\left(f_{1}y-\;f_{2}y\right)}^{2},}$$ (4)

where $h\left(f_{1},f_{2}\right)$ is the Euclidean distance with respect to x and y (Sindhu et al., 2022; Ameur, S. et al., 2020; Sahbani and Adiprawita, 2016; Prakash, K.B. et al., 2020; Alzahrani and Ullah, 2020) for each landmark point of one finger $f_{1}$ with the other landmark of hand $f_{2}$ . Figure 8 shows the graphical representation of n, which is the distance between different landmark points in different hand gestures. If the features that are paired with the Euclidean distance are greater than the specific threshold, then these are distant features defined as Eq. 5.

$$dist\left(f_{1},f_{2}\right)↔d\left(f_{1},f_{2}\right)\geq threshold,$$ (5)

whereas, if the distance between the feature point is smaller than the threshold is adjacent, it is defined as Eq. 6.

$$dist\left(f_{1},f_{2}\right)↔d\left(f_{1},f_{2}\right)\leq threshold.$$ (6)

#### 3.6.2 Full-hand features: polygon meshes

**FIGURE 9:**
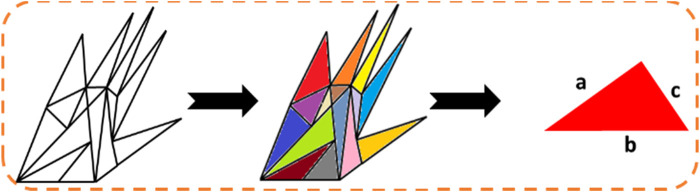
Polygon mesh subdivision into regular triangle shapes.

Polygon meshes is a point-based feature extraction technique. In this method, we have used the palm and finger points obtained from the method discussed in Section 3.4. Hand geometry is formed, which results in different polygon meshes. These polygon shapes vary with the change in the motion of the hand forming different gestures. The polygons formed are irregular polygons generated by combining two or more finger points and palm points. The area in the polygon is computed using Heron’s formula as shown in Figure 9 (Miah, A.S.M. et al., 2023) in Eq. 7.

$$H=\sqrt{t\left(t-a\right)\left(t-b\right)\left(t-c\right)\;}\;where\;t=\frac{a+b+c}{2}.$$ (7)

#### 3.6.3 K-mean ellipsoids

The skeleton, or medial axis, is the set of centroids of the ellipses formed that are tangent to the shape boundary during each hand gesture. The tangent at each pixel point changes where there is a maximum change in the motion of the hand and fingers during the gesture change, where all such ellipses are formed with the boundary of the hand forming a shape. For each ellipsoid, the 16-bin histogram is calculated using the radius. The shape complexity of the hand is defined using the function of entropy in the MAT-based histograms.

The ellipsoids in the circle are denoted by E, and EE represents the fitting within the boundary by tangent and on the skeleton by the augmentative ellipsoid fitting algorithm (AEFA) (Gadekallu, T.R. et al., 2022) Based on GMM-EM models, the ellipsoids evolved by the hypothesis are used to compute the parameters of fixed numbers p when the ellipsoids E get the best coverage within the hand using Eq. 8 (Zhu et al., 2010; Moin, A. et al., 2021; Cha and Vasconcelos, 2008; Chen and Xiang, 2012).

$$A_{i}\left(a\right)=P_{i}\cdot e^{-\left(a-E_{i}\right)SN_{i}\left(a-E_{i}\right)},$$ (8)

where A is the probability of pixels AϵFG , which belong to the ellipsoid $E_{i}$ in our model. $E_{i}$ is the origin of $C_{i},$ whereas $N_{i}$ is the positive definite 2 × 2 matrix representing the orientation and eccentricity of $C_{i}$ . The Gaussian amplitude $P_{i}=1$ ; however, the probability values of $A_{i}\left(a\right)$ on the hand’s boundary are same for all the ellipses. The probability of a point belonging to an ellipse $C_{i}$ is independent of the ellipse size and is dependent on the orientation and position. To get the fixed number of ellipsoids, we have set the value of k = 16.

Algorithm 2 K-mean Ellipsoids.

Input: Binary Image of hands B
Output: Set of ellipsoids E with the lowest AIC [X,Y] = Compute hand shape skeleton (H)
C = compute shape complexity (X,Y)
CC = initialize ellipsoid formation (X, Y)
P = 1
AIC* = ∞
Repeat
H = calculate hypothesis (p, CC)
E = GMM-EM (B, H, P)
AIC = Calculate AIC (B, E, C)
Min_AIC = C.log (1-0.99)+2.k
If AIC < AIC*
Then, AIC* = AIC
E* = E
End
P=P+1
Until P = = 16

**FIGURE 10:**
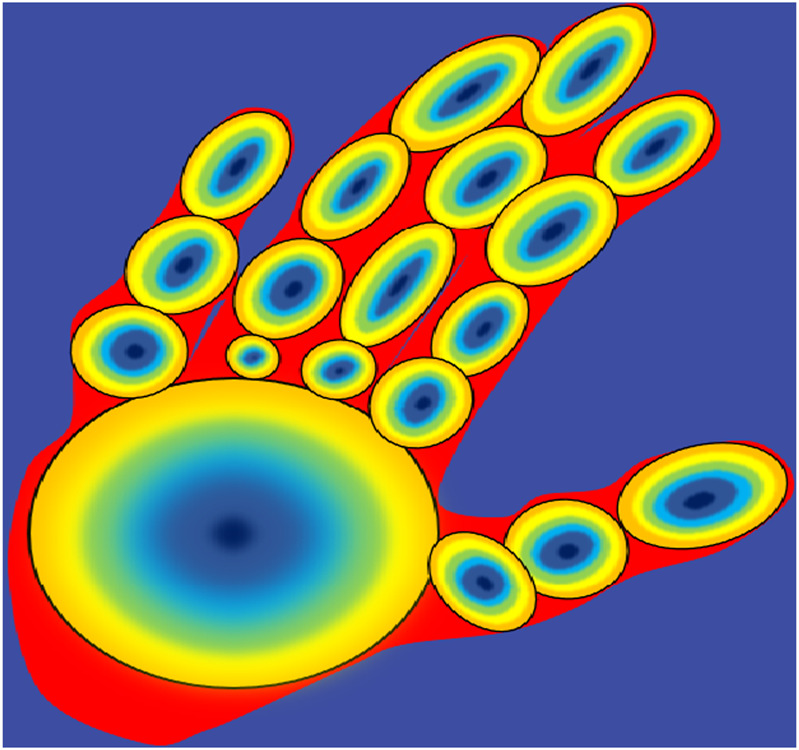
K-mean ellipsoids on hand where k = 16.

Figure 10 shows the results obtained of the ellipsoids formed on the hand in different hand gestures, whereas Algorithm 2 explains the K-mean ellipsoid for feature extraction for hand gesture tracking and recognition system.

#### 3.6.4 Co-occurrence generations

After the extraction of all point-based features, the co-occurrence matrix (Li et al., 2022) is computed. The purpose of using this technique is the distribution of the co-occurring point-based features at a given offset and save the execution time to improve the efficiency of our model defined as Eq. 9.

$$M_{i,j}=\frac{1}{N}\sum_{g=1}^{G}\delta \left(Y_{1}i,Y_{2}j\right),$$ (9)

where $Y_{1}i$ is the *i*th cue value of the first finger and $Y_{2}j$ is the *j*th cue of the second finger or palm of the same image (Rabiee.H. et al., 2016). Such means are beneficial to improve the overall efficiency of the recognition system, especially essential for a future real-time application potency (Liu and Schultz, 2018; Liu, H. et al., 2023).

### 3.7 Particle swarm optimization (PSO)

**FIGURE 11:**
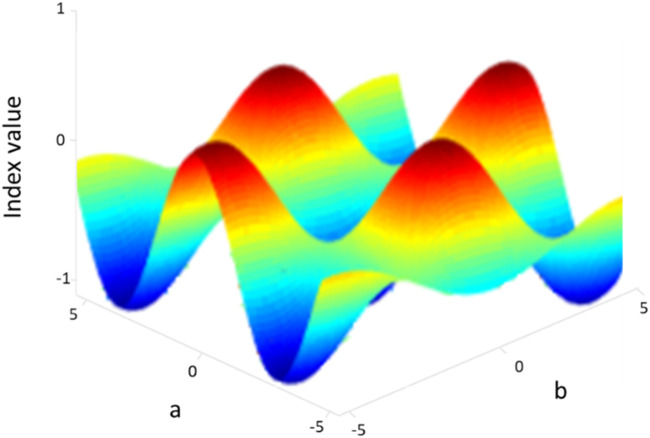
Particle swarm optimization over the ISL dataset.

After the successful extractions of the features, we have applied the particle swarm optimization algorithm (Figure 11; Chriki and Kamoun, 2021) to get the optimal set of features. In this method, each feature is considered a particle. A number of iterations are performed, and after every iteration, an updated optimized swarm of particles is achieved. PSO randomly initializes the swarm of particles and acts on their social behavior. Thus, to find out the most optimum particles, PSO adjusts each particle trajectory toward its own location and to the global best particle in the swarm. This is done using the following equations (Eqs 10, 11a) (Abdulhussain, S.H. et al., 2019) and Algorithm 2.

$$p_{best}\left(a,b\right)=\arg\min_{k=1,2,\ldots ,b}\left[f\left(Q_{s}\left(k\right)\right)\right],\;s\epsilon \left\{1,2,3,\ldots ,N_{p}\right\},$$ (10)

$$gbest\left(b\right)=\arg\min_{s=1,2,\ldots ,N_{p}}\left[f\left(Q_{s}\left(k\right)\right)\right],\;k\epsilon \left\{1,2,3,\ldots ,b\right\},$$ (11a)

where $N_{p}$ denotes the total number of particles, s denotes the particle index, b is the current iteration, f is the fitness function, and Q is the position of the particle (Ma and Li, 2018; Miao and Zhang, 2019; Xu and Xu, 2016). Figure 11 shows the optimization graph.

#### 3.7.1 One-dimensional CNN

**FIGURE 12:**
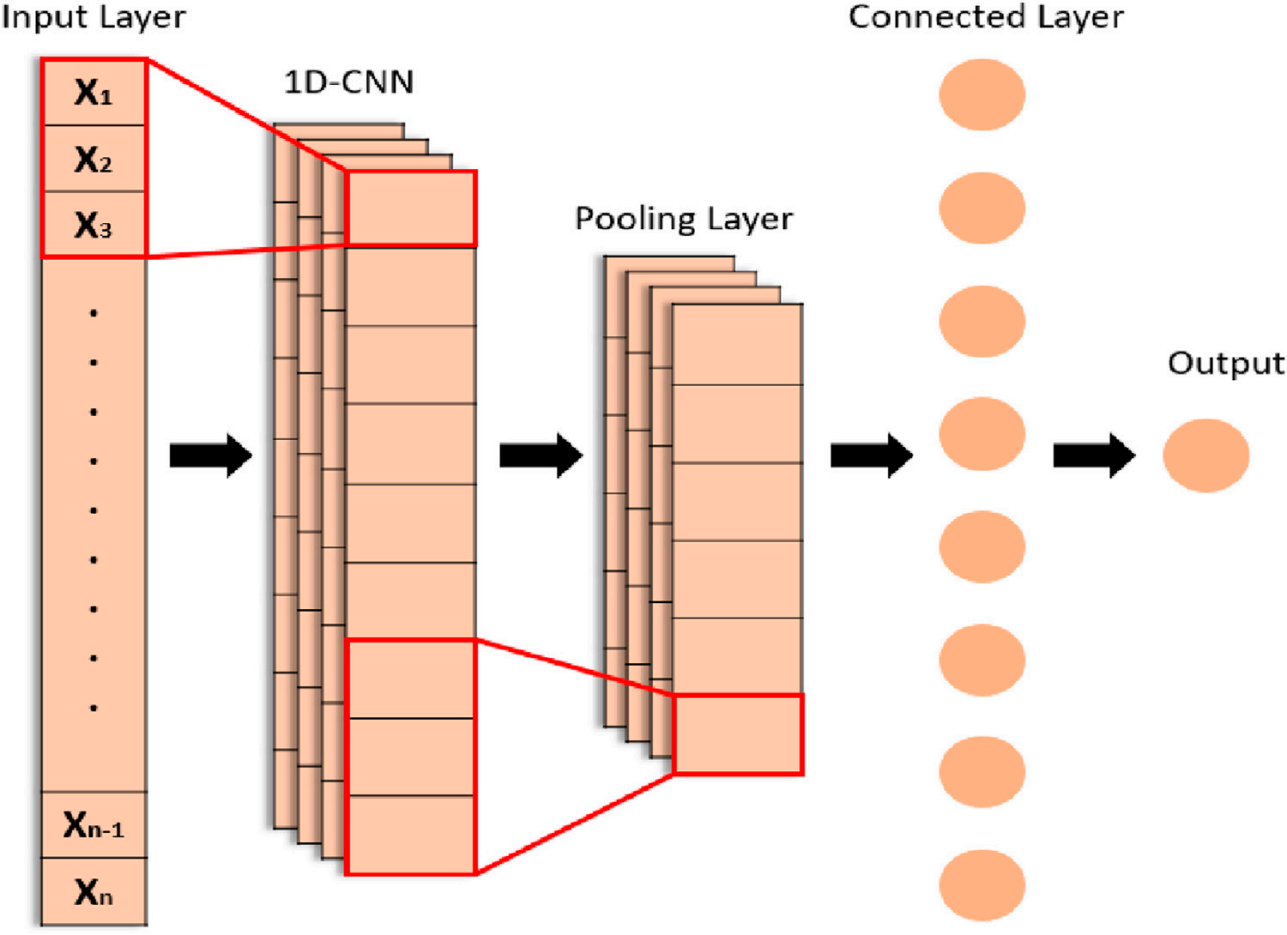
Architecture of the 1D CNN model.

All the point-based features extracted from the abovementioned techniques are then passed through the CNN, which results in the classification of hand gestures. It is observed in many research studies that the CNN is powerful in the classification of images and video-based features (Saqib et al., 2019; Pandey et al., 2020; Reddy and Wang, 2020) than other deep learning techniques. Figure 12 illustrates the overall architecture of our proposed 1D CNN for hand gesture tracking and recognition.

In our model, we have used 1D CNN for the first time in hand gesture tracking and recognition for learning and medical staff assistance. The ISL dataset contains 9,876 feature sets of videos. Our proposed 1D CNN contains three convolution layers, three max-pooling layers, and one fully connected layer. First convolution layer $L_{1}$ contains the input matrix. This layer is convolved with 32 kernels. Each layer having a size of 1×13 which as a result produced a matrix of 4500×10488×32 . The convolution matrix is calculated as done in Eqs 11b and 12.

$$L_{n}^{m-1}\left(x,y\right)=ReLU\left(z\right),$$ (11b)

$$ReLU\left(z\right)=\sum_{u=1}^{y}\Omega \left(a,\left(b-u+\frac{y+1}{2}\right)\right){weight}_{n}^{m}\left(u\right)+\alpha_{n}^{m},$$ (12)

where $L_{n}^{m-1}\left(x,y\right)$ denotes the convolution layer result for the two coordinates x and y of the m-1 layer with the *n*th convolution map. The size of the kernel is represented by z, and the previous layer map is represented by ${weight}_{n}^{m}$ is the *m*th convolution kernel for the layer n, whereas $\alpha_{n}^{m}$ is the *m*th bias of the n kernel. The result produced by the first convolution layer is passed to the next max-pooling layer $M_{1}$ . A ReLU is used between the convolution and max pooling layers. It is responsible for passing the previous layer weights and bias to the next layer (He and Gong, 2021; Neiswanger and Xing, 2014; Li et al., 2022). The max-pooling layer downsamples the resulted matrix produced from the convolution layer by using a sliding window of 1×2 . The pooling results are calculated as using Eq. 13.

$$S_{n}^{m-1}\left(a,b\right)=\max\;(L_{n}^{m}\left(a,\left(\left(b-1\right)\times \left(p+q\right)\right)\right),$$ (13)

where 1≤p≤q and n denotes the pooling window size. The first pooling layer results are passed to the second convolution layer $L_{2}$ that is convolved with 64 kernels and is passed to the next max pooling layer $M_{2}$ . The same practice is followed by the next layer that is convolved with 128 kernels. At the end, a fully connected layer is obtained defined as Eq. 14.

$$F_{n}^{m+1}=ReLU\left(\sum_{i}x_{i}^{m}{weight}_{mv}^{m}+\alpha_{v}^{m}\right).$$ (14)

**FIGURE 13:**
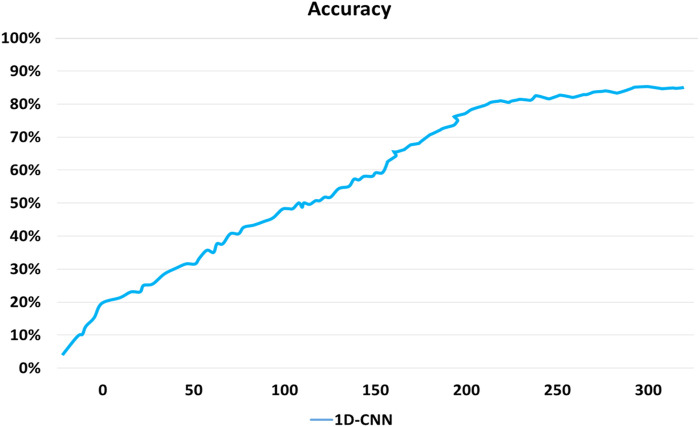
Performance evaluation of the 1D CNN model.

From the above equation, ${Weight}_{iv}^{m}{weight}_{iv}^{m}$ is the matrix having weights from the node i of layer m to the node v of layer m + 1. $x_{i}^{m}$ denotes the node m content at layer i. Figure 13 represents the convergence plot of 1D CNN of all datasets using 300 epochs.

## 4 System validation and experimentation

### 4.1 Experimental setup

In this section, we have discussed the experiments performed to validate our proposed model. The backend of the system is developed in Python using Visual Studio Code. The hardware system used is Intel Core i5-6200U with 2.40 GHz processing power, 16 GB RAM, 2 GB dedicated graphics card Nvidia 920M having x64 based Windows 10 pro. We have divided this section into three subsections. In the first Section 4.1, we have discussed the details of the benchmark datasets used in our proposed system. In Section 4.2, we tested our model using various performance metrics.

### 4.2 Dataset description

The ISL and WLASL datasets were the two that we used in our investigation. A variety of hand gestures used in Indian sign language communication can be found in the ISL dataset, which is a compilation of gestures from the Indian Sign Language (ISL). In contrast, the American Sign Language (ASL) hand motions found in the WLASL dataset are commonly utilized in sign language recognition studies.

We have access to the ISL dataset at [https://live.european-language-grid.eu/catalogue/lcr/7631], as it is accessible to the public. Additionally, accessible to the general public, the WLASL dataset can be found at [https://www.kaggle.com/risangbaskoro/WLASL-Processed].

#### 4.2.1 ISL dataset

The ISL dataset contains video files of the eight hand gestures (Sharma and Singh, 2021). The gestures include accident, call, doctor, help, hot, lose, pain, and thief. The dataset is specially designed for emergency situations. The videos have been collected from 26 individuals including 12 men and 14 women between the age group of 22–26 years. The videos are captured indoor under normal lighting conditions by placing the camera at a fixed distance.

#### 4.2.2 WLASL dataset

The WLASL dataset is the largest video dataset of the ASL hand gesture dataset (Li, D. et al., 2020). It contains 2000 hand gesture classes. The dataset is especially designed for communication between the deaf and hearing communities. We have taken eight classes used for the communication between the teachers and the students, i.e., hungry, wish, scream, forgive, attention, appreciate, abuse, and admit.

## 5 Results and analysis

We provide a thorough analysis of our suggested hand gesture recognition system in this section. We used a variety of performance indicators to test the system’s efficacy on the ISL and WLASL datasets. Initially, we computed our system’s accuracy, which expresses the total percentage of correctly classified data. Using the ISL and WLASL datasets, our system’s accuracy was found to be 83.71% and 85.71%, respectively. We also calculated precision, recall, and F1-score for every hand gesture class to give a more thorough study. Recall gauges the percentage of real cases correctly identified for a given class, while precision shows the percentage of correctly classified instances inside that class. The F1-score integrates both recall and precision into a single metric. For most hand gesture classes, our system’s precision, recall, and F1-score values were favorable, indicating that it can distinguish various motions with effectiveness. In addition, we evaluated the misclassification rate, which is the proportion of cases that were erroneously classified. We found that the misclassification rate differed among hand gesture classes, underscoring the difficulties in recognizing gestures, especially when they have identical visual characteristics.

Apart from the quantitative assessment, we conducted a qualitative analysis of the outcomes. We looked at examples of hand gestures that were identified properly and erroneously in order to look into possible causes of misclassifications. We were able to pinpoint areas that needed improvement and gain understanding of the system’s functionality, thanks to this qualitative investigation.

### 5.1 Hand gesture detection and recognition accuracy

Supplementary Tables S1, S2 show the overall detection accuracies concerning different video frames sequences over the ISL and WLASL datasets for this study.

### 5.2 Confusion matrix of the proposed hand gesture recognition

To measure the performance of our system, we have used the confusion matrix of two datasets shown in Supplementary Tables S3, S4.

### 5.3 Other performance measures of our proposed model

We have used five evaluation metrics, i.e., precision (Eq. 16), recall (Eq. 17), F1-score (Eq. 18), accuracy (Eq. 19), and misclassification rate (Eq. 20), using the following equations, whereas Supplementary Tables S5, S6 show the results of all these evaluation metrics over three benchmark datasets.

$$\text{Precision}=\frac{TP\;}{TP+FP},$$ (16)

$$\text{Recall}=\frac{TP\;}{TP+FN},$$ (17)

$$F1\text{ score}=2\times \left(\frac{Precision\times Recall\;}{Precision+Recall}\right),$$ (18)

$$\text{Accuracy}=\frac{TP+TN\;}{TP+TN+FP+FN},$$ (19)

$$\text{Misclassification}=\frac{FP+FN\;}{TP+TN+FP+FN},$$ (20)

where TP denotes true positive, TN is true negative, FP is false positive, and FN is false negative.

### 5.4 Comparison of 1D CNN with other well-known classifiers

**FIGURE 14:**
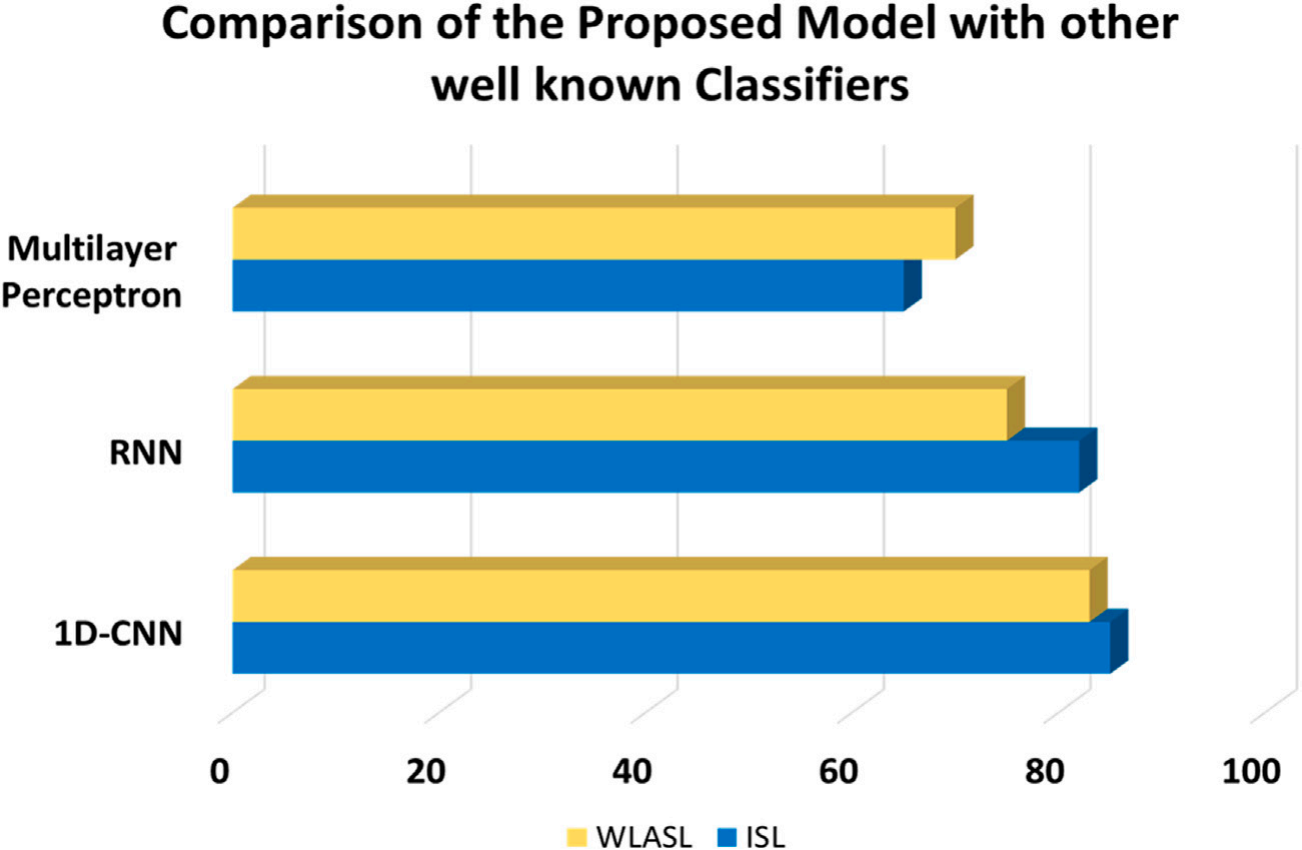
Comparison graph of the proposed model with other well-known classifiers.

In this experiment, we have compared the hand gesture tracking and recognition results with other state-of-the-art models. It is observed from our experiment that 1D CNN gives more precise and accurate results of hand gesture tracking and recognition. Figure 14 shows the comparison graph of our proposed model with other well-known classifiers.

In operating rooms, doctors could operate computer interfaces, change settings, and operate medical equipment with hand gestures without compromising the sterile environment. Diagnostic imaging: by using hand gestures to control medical imaging software (such as zoom, pan, and rotate), radiologists and technicians can free up their hands for other duties.

Some possibly helpful hand gestures based on the healthcare use cases include pinch-to-zoom: to zoom in or out of computer displays or medical pictures, pinch and spread your fingers. Swipe/scroll: use hand gestures to swipe through slide shows or patient records. Point/choose: making pointing motions to highlight particular regions on a screen or to choose alternatives. Rotation: rotate 3D medical models or change the orientation of surgical instruments with hand motions that twist. Volume control involves raising and lowering the hand to adjust the sound of warnings or instructions.

Limitations: different hand shapes and sizes: the system might need to be resilient to variations in skin tones, hand sizes, and other physical features. Blockages and occlusions: preserving precise hand tracking and gesture recognition while medical equipment, surgical gowns, and other things are present. Lighting conditions: guaranteeing dependable performance in a range of healthcare environments, such as operating rooms and exam rooms, with varying lighting requirements.

### 5.5 Comparison of the proposed model with other conventional methods

**FIGURE 15:**
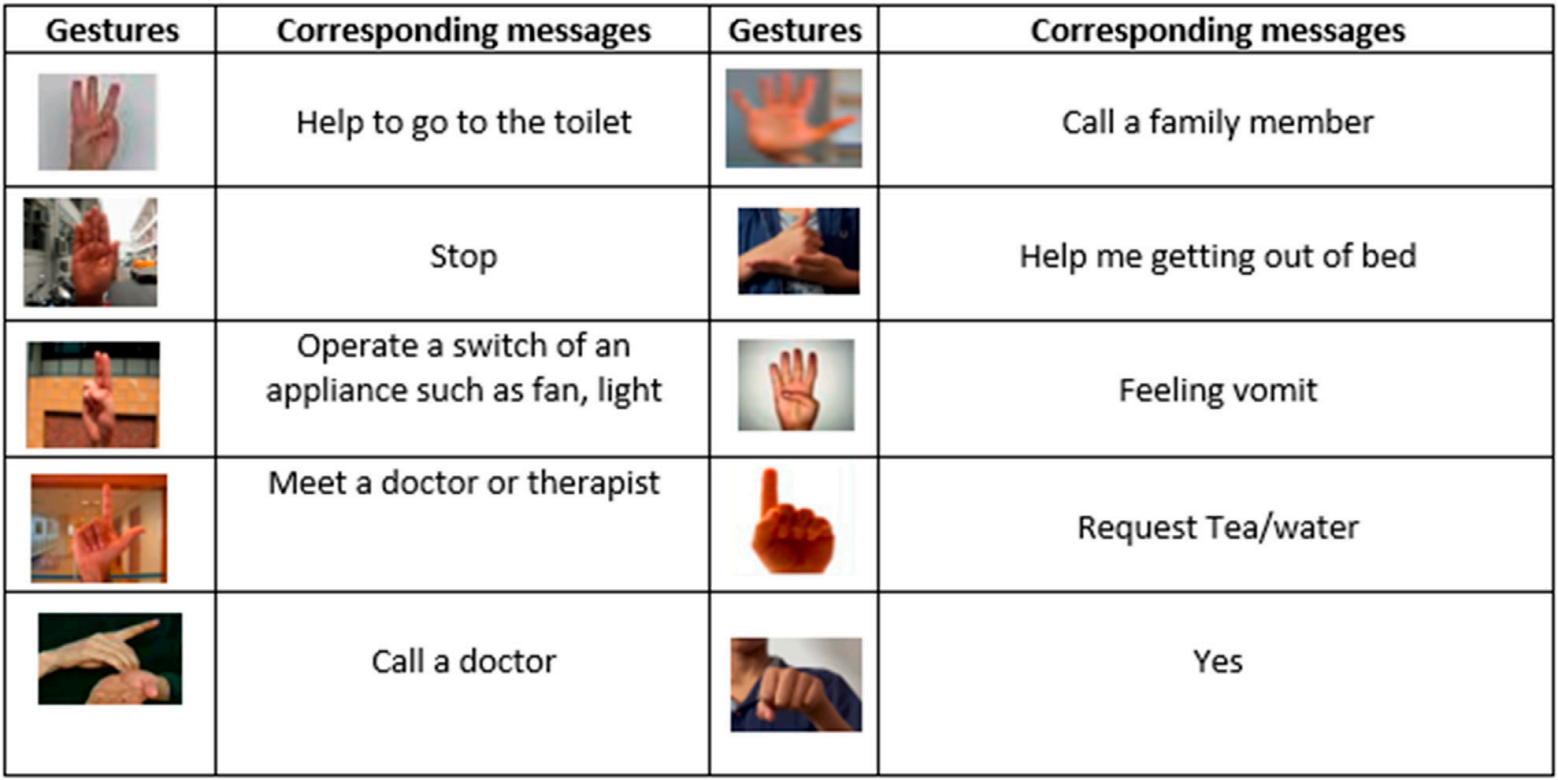
The figure is showing some hand gestures with their corresponding messages. Gestures and their corresponding message.

In this experiment, we have compared the proposed hand gesture tracking and recognition model with conventional models as shown in Figure 15 (Kumar and Kumar, 2020). After extensive testing on all 26 alphabet signs, their algorithm achieved an astounding 100% accuracy on the majority of them. The average accuracy for all alphabet signs was an astounding 80.76%, even with these anomalies. These outcomes show the system’s good performance and its capacity to correctly identify and categorize most letter signs. Hosain, A.A. et al. (2021) offers a unique pose-guided pooling technique that improves the extraction of additional features from a 3D CNN within the context of global sign language recognition. They get notable gains in overall recognition accuracy (68.30%) on the WLASL 300 dataset by incorporating features from several network layers. This study (Sharma, S. et al., 2021) presents the G-CNN deep learning model for classifying hand gestures in sign language. The model outperforms state-of-the-art methods with high accuracy (94.83%, 99.96%, and 100%) across many gesture categories. Because it does away with user reliance and the requirement for external hardware, the G-CNN approach is useful. It functions well with enhanced data and is resilient to scaling and rotation changes.

Our proposed system provides promising results with the techniques (combination of machine learning algorithm and CNN) used in our model. Supplementary Table S7 provides the gesture tracking and recognition accuracies over the ISL and WLASL datasets with other state-of-the-art methods.

## 6 Discussion and limitations

Considering the healthcare scenario, the objective of this work is to create a dependable and efficient system that can understand patient hand gestures in order to improve communication between patients and healthcare providers in the healthcare setting. Our goal is to train a reliable gesture recognition system that can function well in a range of healthcare environments by utilizing publically available hand gesture recognition image datasets. There are seven classes of datasets in NUS. NUS I is not included because the images in it have uniform backgrounds. However, NUS II has images that show every challenge that arises while recognizing hand gestures. Two thousand color images and 700 images depicting human skin in regions other than the hands are included. We evaluate our proposed system that interprets the hand gestures made by the patient and transmits messages to healthcare professionals. The purpose we had in developing this hand gesture recognition system is to improve communication between patients and healthcare providers in environments where verbal communication may be difficult or limited. This will allow patients to communicate their needs, concerns, or messages to the staff more effectively.

The system might need to be resilient to variations in skin tones, hand sizes, and other physical features. When there are a lot of skin objects (many hands or faces) available in the background, the system usually performs poorly. The only skin thing the system is likely to pick up in a hospital setting, though, is the patient’s hand. However, we will continue to strive for improved efficiency when there are several skin objects present, guaranteeing dependable performance in a range of healthcare environments, such as operating rooms and exam rooms, with varying lighting requirements.

## 7 Conclusion

We have presented a novel method for hand gesture recognition in the fields of medicine and e-learning in this article. We employ pre-processing RGB frames, backdrop modeling, and CNN blob detection methods for hand movement detection in our methodology. For skeleton mapping, we have implemented the SSMD approach, and point- and texture-based features are retrieved according to our earlier studies. We have used the PSO algorithm to optimize the characteristics. Lastly, a 1D CNN is used to classify hand gestures. After a great deal of experimentation, we have obtained encouraging findings. It is important to recognize the limitations of our system, though. In particular, our model’s classification accuracy declines with similar-looking hand gestures. Furthermore, the inability to clearly see fingers impairs the precision of skeleton mapping.

Future studies will concentrate on resolving these issues and simplifying the system. We also intend to further our research by investigating additional hand gesture classifications for the medical and e-learning domains.

## Data Availability

The original contributions presented in the study are included in the article/Supplementary Material; further inquiries can be directed to the corresponding author.
