# Supplementary material for: Innovative healthcare solutions: robust hand gesture recognition of daily life routines using 1D CNN
Source: Front Bioeng Biotechnol. 2024 Jul 31;12:1401803. doi: 10.3389/fbioe.2024.1401803 (PMC11322365; doi:10.3389/fbioe.2024.1401803)
Supplement: Supplementary file 1 [file Table1.docx]

Table 1. Hand Gestures Tracking and Recognition Accuracy over ISL dataset

| **Sequence No**  **(Frame = 60)** | **Actual Tracking** | **Successful** | **Failure** | **Accuracy** |
| --- | --- | --- | --- | --- |
| 8 | 5 | 5 | 0 | 1 |
| 16 | 4 | 3 | 1 | 0.75 |
| 24 | 4 | 4 | 0 | 1 |
| 32 | 3 | 3 | 0 | 1 |
| 40 | 4 | 3 | 3 | 0.75 |
| 48 | 4 | 2 | 2 | 0.5 |
| 56 | 4 | 4 | 0 | 1 |
| **Mean Accuracy=85.71%** | | | | |
