# Supplementary material for: Innovative healthcare solutions: robust hand gesture recognition of daily life routines using 1D CNN
Source: Front Bioeng Biotechnol. 2024 Jul 31;12:1401803. doi: 10.3389/fbioe.2024.1401803 (PMC11322365; doi:10.3389/fbioe.2024.1401803)
Supplement: Supplementary file 2 [file Table8.docx]

| **TARGET** | **Hungry** | **Help** | **Toilet** | **Switch** | **Doctor** | **Family** | **Stop** | **Vomit** | **SUM** |
| --- | --- | --- | --- | --- | --- | --- | --- | --- | --- |
| **Hungry** | 80 | 0 | 0 | 8 | 6 | 2 | 0 | 4 | 80% |
| **Help** | 0 | 84 | 4 | 4 | 2 | 0 | 6 | 0 | 84% |
| **Toilet** | 1 | 0 | 87 | 2 | 2 | 6 | 0 | 2 | 87% |
| **Switch** | 0 | 1 | 5 | 89 | 0 | 0 | 2 | 3 | 89% |
| **Doctor** | 3 | 0 | 0 | 0 | 86 | 8 | 0 | 3 | 86% |
| **Family** | 0 | 0 | 3 | 5 | 1 | 85 | 5 | 0 | 85% |
| **Stop** | 0 | 2 | 0 | 1 | 0 | 08 | 86 | 3 | 86% |
| **Vomit** | 4 | 0 | 0 | 2 | 0 | 4 | 10 | 80 | 80% |
| **SUM** | 88  90.9% | 87  96.5% | 99  87.8% | 102  86.5% | 97  87.2% | 113  75..3% | 109  81.9% | 95  84.2% | 677/800  84.6% |

Table 8. Confusion matrix for hand gesture recognition accuracy over NUS dataset
