# Supplementary material for: Innovative healthcare solutions: robust hand gesture recognition of daily life routines using 1D CNN
Source: Front Bioeng Biotechnol. 2024 Jul 31;12:1401803. doi: 10.3389/fbioe.2024.1401803 (PMC11322365; doi:10.3389/fbioe.2024.1401803)
Supplement: Supplementary file 3 [file Table7.docx]

Table 7. Comparison of gesture tracking and recognition accuracy over ISL and WLASL dataset with other-state-of-the-art-methods

| **Method** | **ISL Dataset (%)** | **Method** | **WLASL Dataset (%)** |
| --- | --- | --- | --- |
| **( Raghuveera, T. et al.,2020)** | 71.85 | **(Li, D. et al., 2020)** | 62.63 |
| **(Bhaga, N.K. et al., 2019)** | 78.3 | **(Hosain, A.A. et al., 2021)** | 66.7 |
| **(Jayadeep,G. et al., 2020)** | 81 | **(Dafnis, K.M. et al.,2022)** | 77.43 |
| **(Kumar et al, 2020)** | 80.76 | **-** | - |
| **Proposed** | **85.7** | **Proposed** | **83.7** |
