# Supplementary material for: Innovative healthcare solutions: robust hand gesture recognition of daily life routines using 1D CNN
Source: Front Bioeng Biotechnol. 2024 Jul 31;12:1401803. doi: 10.3389/fbioe.2024.1401803 (PMC11322365; doi:10.3389/fbioe.2024.1401803)
Supplement: Supplementary file 5 [file Table5.docx]

Table 5. Evaluation metrics results over ISL dataset

| **Class Name** | **Precision** | **1-Precision** | **Recall** | **1-Recall** | **F1-score** |
| --- | --- | --- | --- | --- | --- |
| **Accident** | 0.86 | 0.14 | 0.93 | 0.07 | 0.90 |
| **Call** | 0.87 | 0.13 | 0.82 | 0.18 | 0.84 |
| **Doctor** | 0.85 | 0.15 | 0.89 | 0.14 | 0.87 |
| **Help** | 0.87 | 0.13 | 0.81 | 0.19 | 0.84 |
| **Hot** | 0.88 | 0.12 | 0.87 | 0.13 | 0.88 |
| **Lose** | 0.86 | 0.14 | 0.86 | 0.14 | 0.86 |
| **Pain** | 0.80 | 0.20 | 0.83 | 0.17 | 0.82 |
| **Thief** | 0.87 | 0.13 | 0.86 | 0.14 | 0.87 |
| **Accident** | 0.86 | 0.14 | 0.93 | 0.07 | 0.90 |
| **Accuracy** | | | **0.857** | | |
| **Misclassification Rate** | | | **0.143** | | |
| **Weighted-F1** | | | **0.857** | | |
