# Supplementary material for: Innovative healthcare solutions: robust hand gesture recognition of daily life routines using 1D CNN
Source: Front Bioeng Biotechnol. 2024 Jul 31;12:1401803. doi: 10.3389/fbioe.2024.1401803 (PMC11322365; doi:10.3389/fbioe.2024.1401803)
Supplement: Supplementary file 6 [file Table3.docx]

Table 3. Confusion matrix for hand gesture recognition accuracy over ISL dataset

| **TARGET** | **Accident** | **Call** | **Doctor** | **Help** | **Hot** | **Lose** | **Pain** | **Thief** | **SUM** |
| --- | --- | --- | --- | --- | --- | --- | --- | --- | --- |
| **Accident** | 86 | 2 | 0 | 0 | 2 | 0 | 10 | 0 | 86% |
| **Call** | 0 | 87 | 0 | 11 | 0 | 0 | 0 | 2 | 87% |
| **Doctor** | 2 | 0 | 85 | 0 | 0 | 9 | 4 | 0 | 85% |
| **Help** | 0 | 8 | 0 | 87 | 2 | 0 | 0 | 3 | 87% |
| **Hot** | 1 | 0 | 6 | 0 | 88 | 3 | 2 | 0 | 88% |
| **Lose** | 2 | 0 | 2 | 1 | 0 | 86 | 0 | 9 | 86% |
| **Pain** | 0 | 9 | 0 | 9 | 0 | 2 | 80 | 0 | 80% |
| **Thief** | 1 | 0 | 3 | 0 | 9 | 0 | 0 | 87 | 87% |
| **SUM** | 92  93.4% | 106  82.1% | 96  88.5% | 108  80.55% | 101  87.1% | 100  86% | 96  83.3% | 101  86.1% | 686/800  85.7% |
