# Supplementary material for: Innovative healthcare solutions: robust hand gesture recognition of daily life routines using 1D CNN
Source: Front Bioeng Biotechnol. 2024 Jul 31;12:1401803. doi: 10.3389/fbioe.2024.1401803 (PMC11322365; doi:10.3389/fbioe.2024.1401803)
Supplement: Supplementary file 7 [file Table4.docx]

| **TARGET** | **Hungry** | **Wish** | **Scream** | **Forgive** | **Attention** | **Appreciate** | **Abuse** | **Admit** | **SUM** |
| --- | --- | --- | --- | --- | --- | --- | --- | --- | --- |
| **Hungry** | 82 | 0 | 0 | 8 | 6 | 0 | 0 | 4 | 82% |
| **Wish** | 0 | 87 | 4 | 0 | 2 | 0 | 7 | 0 | 87% |
| **Scream** | 8 | 0 | 82 | 2 | 0 | 6 | 0 | 2 | 82% |
| **Forgive** | 0 | 8 | 0 | 84 | 2 | 0 | 6 | 0 | 84% |
| **Attention** | 3 | 0 | 0 | 0 | 80 | 8 | 0 | 9 | 80% |
| **Appreciate** | 0 | 0 | 0 | 0 | 5 | 85 | 10 | 0 | 85% |
| **Abuse** | 0 | 2 | 0 | 1 | 0 | 3 | 86 | 8 | 86% |
| **Admit** | 9 | 0 | 0 | 2 | 0 | 0 | 5 | 84 | 84% |
| **SUM** | 102  80.4% | 97  89.7% | 86  95.3% | 97  86.5% | 95  84.2% | 102  83.3% | 114  75.4% | 107  78.5% | 670/800  83.7% |

Table 4. Confusion matrix for hand gesture recognition accuracy over WLASL dataset
