# Supplementary material for: Innovative healthcare solutions: robust hand gesture recognition of daily life routines using 1D CNN
Source: Front Bioeng Biotechnol. 2024 Jul 31;12:1401803. doi: 10.3389/fbioe.2024.1401803 (PMC11322365; doi:10.3389/fbioe.2024.1401803)
Supplement: Supplementary file 8 [file Table6.docx]

Table 6. Evaluation metrics results over WLASL dataset

| **Class Name** | **Precision** | **1-Precision** | **Recall** | **1-Recall** | **F1-score** |
| --- | --- | --- | --- | --- | --- |
| **Hungry** | 0.82 | 0.18 | 0.80 | 0.20 | 0.81 |
| **Wish** | 0.87 | 0.13 | 0.90 | 0.14 | 0.88 |
| **Scream** | 0.82 | 0.18 | 0.95 | 0.05 | 0.88 |
| **Forgive** | 0.84 | 0.16 | 0.87 | 0.13 | 0.85 |
| **Affiliation** | 0.80 | 0.20 | 0.84 | 0.16 | 0.82 |
| **Appreciate** | 0.85 | 0.15 | 0.83 | 0.17 | 0.84 |
| **Abuse** | 0.86 | 0.14 | 0.75 | 0.25 | 0.80 |
| **Admit** | 0.84 | 0.16 | 0.79 | 0.21 | 0.81 |
| **Accuracy** | | | **0.837** | | |
| **Misclassification Rate** | | | **0.163** | | |
| **Weighted-F1** | | | **0.837** | | |
